# Supplementary figures and images for: Phosphorylated histone variant γH2Av is associated with chromatin insulators in Drosophila
Source: PLoS Genet. 2022 Oct 5;18(10):e1010396. doi: 10.1371/journal.pgen.1010396 (PMC9576066; doi:10.1371/journal.pgen.1010396)

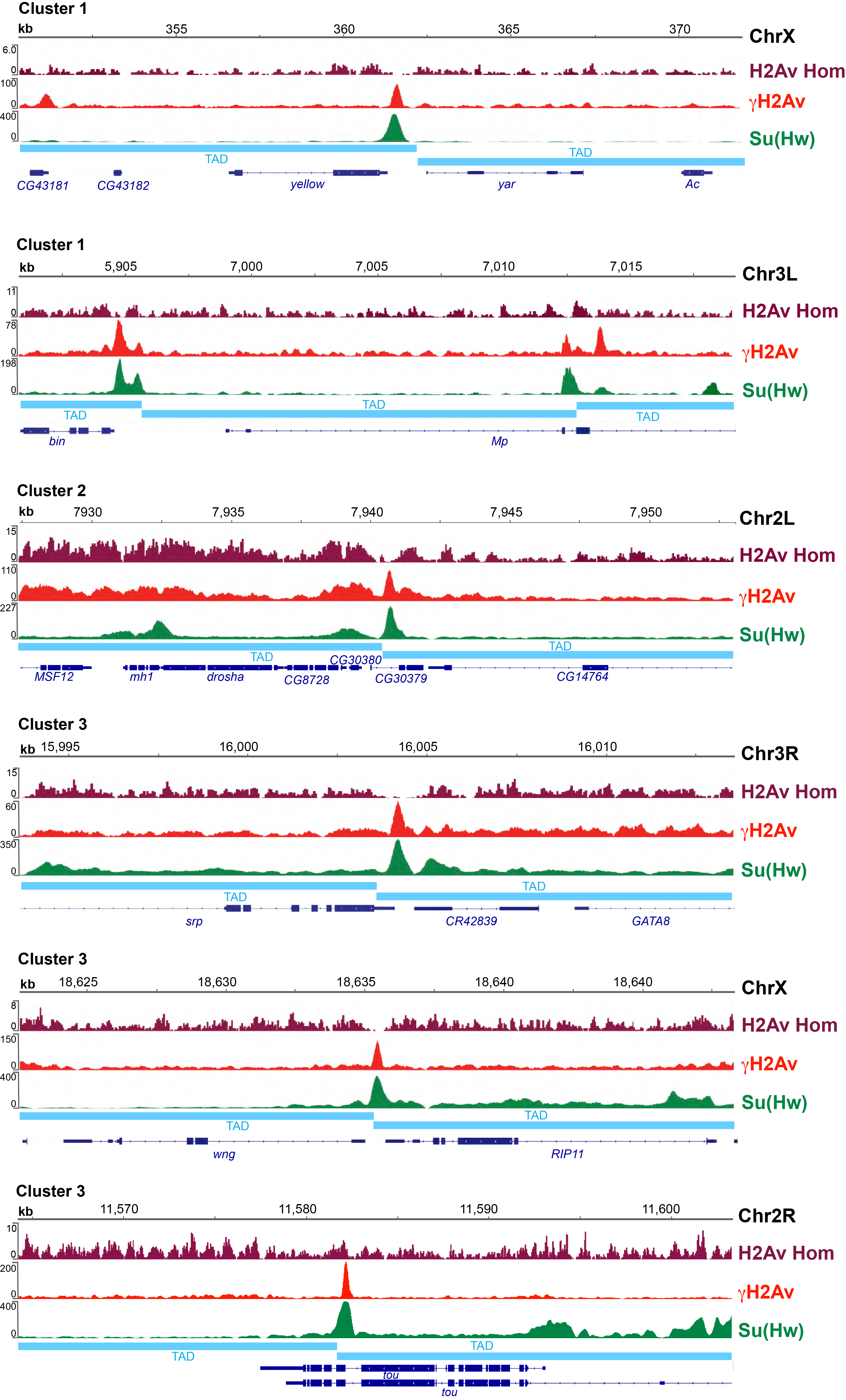

Supplement: S1 Fig — Profiles include examples from all chromosomes and clusters, as defined in Fig 3. (TIF) [file pgen.1010396.s001.tif]

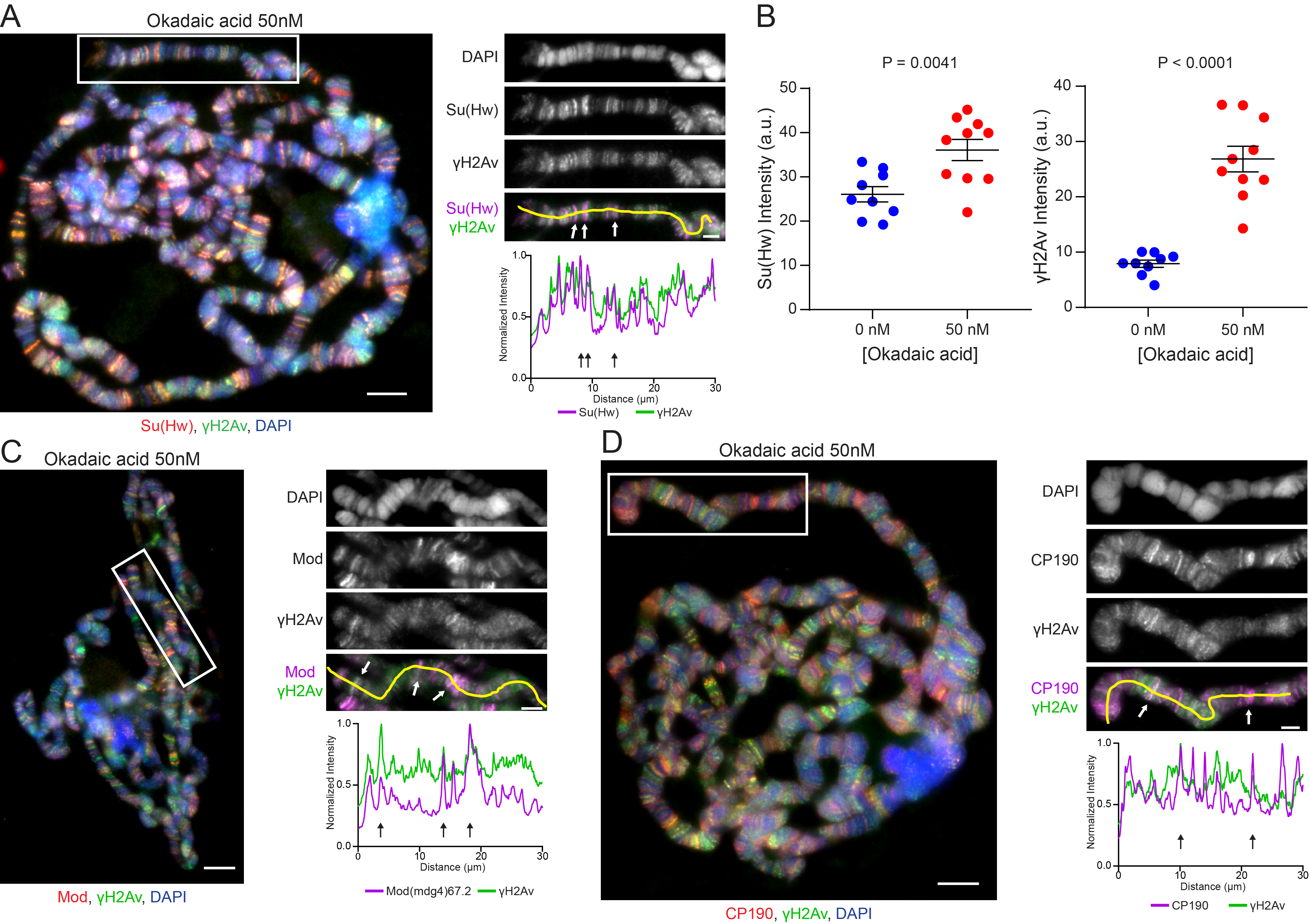

Supplement: S2 Fig — Shown are co-immunostains of polytene chromosomes from salivary glands treated with okadaic acid. A. Immunostaining of γH2Av with Su(Hw). B. Quantification of the immunostaining data show in A. The intensities of Su(Hw) and γH2Av are shown bellow, in the absence and presence of okadaic acid. Each point represents the polytene genome of an individual cell. Error bars represent one standard error of the mean. P-values were determined using unpaired two-tailed Student’s T-tests. C. Immunostaining of γH2Av with Mod(mdg4)67.2. D. Immunostaining of γH2Av with CP190. Immunofluorescent micrographs of polytene chromosome squashes are shown on the left. Magnified insets are shown to the right of each figure, corresponding to the white boxes in the figures on the left. Scale bars are 5 μm in the figures and 2 μm in the insets. Insets are shown as RGB merge, with DAPI on the blue channel, γH2Av on the green channel, and various insulator proteins on the red channel. Red and green channels are shown independently in grey scale and merged as magenta and green. Beneath the insets are linescans corresponding to the yellow lines in the merged insets. Linescan intensities in A, C, and D were normalized by dividing each value by the maximum intensity recorded on each channel. (TIF) [file pgen.1010396.s002.tif]

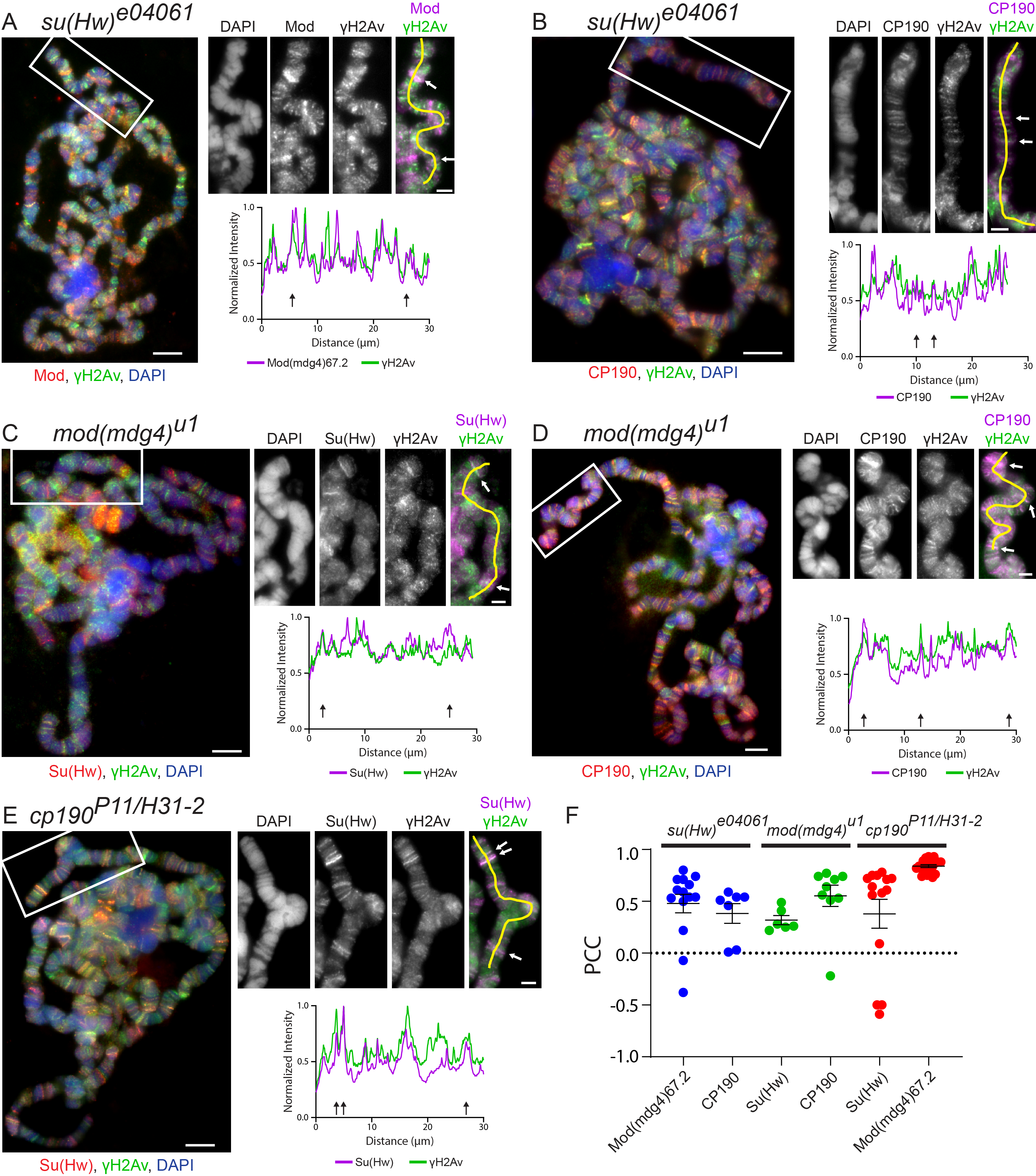

Supplement: S3 Fig — Shown are co-immunostains of polytene chromosomes from salivary glands treated with okadaic acid. A. Colocalization of γH2Av with Mod(mdg4)67.2 in su(Hw)e04061. B. Colocalization of γH2Av with CP190 in su(Hw)e04061. C. Colocalization of γH2Av with Su(Hw) in mod(mdg4)u1. D. Colocalization of γH2Av with CP190 in mod(mdg4)u1. E. Colocalization of γH2Av with Su(Hw) in cp190P11/H31-2. F. Pearson’s Correlation Coefficient (PCC) for γH2Av signal with each insulator protein signal is plotted, with each point representing the polytene genome of each cell. Error bars represent one standard error of the mean. PCC values are grouped by genotype (red = su(Hw)e04061, green = mod(mdg4)u1, blue = cp190P11/H31-2). Immunostaining results from polytene chromosome squashes are shown on the left in each panel. Magnified insets are shown to the right of each figure, corresponding to the white boxes in the figures on the left. Scale bars are 5 μm in the figures and 2 μm in the insets. Insets are shown as RGB merge, with DAPI on the blue channel, γH2Av on the green channel, and various insulator proteins on the red channel. Red and green channels are shown independently in grey scale and merged as magenta and green. Beneath the insets are linescans corresponding to the yellow lines in the merged insets. Linescan intensities were normalized by dividing each value by the maximum intensity recorded on each channel. (TIF) [file pgen.1010396.s003.tif]
