# Supplementary material for: Phosphorylated histone variant γH2Av is associated with chromatin insulators in Drosophila
Source: PLoS Genet. 2022 Oct 5;18(10):e1010396. doi: 10.1371/journal.pgen.1010396 (PMC9576066; doi:10.1371/journal.pgen.1010396)
Supplement: Attachment — Submitted filename: response to reviewers R commons PG .pdf [file pgen.1010396.s005.pdf]

final review version to Initial reviews and responses from Review commons:

Date:

2022-02-28 06:07:42

Last Sent:

2022-02-28 06:07:42

Triggered By:

Redacted

BCC:

Redacted

Subject:

Review Commons Reviews for Refereed Preprint RC-2021-01222

Message:

February 28, 2022

RE: Review Commons Refereed Preprint #RC-2021-01222

Dr. Mariano Labrador
The University of Tennessee
Biochemistry & Cell and Molecular Biology
309 Ken and Blaire Mossman Bldg.
1311 Cumberland Avenue
Knoxville, Tennessee 37996

Dear Dr. Labrador,

Thank you for sending your manuscript entitled "Phosphorylated Histone variant γH2Av is associated with Chromatin Insulators in Drosophila" to Review Commons. We have now completed the peer review of the manuscript. Please find the full set of reports below.

Note: in case you have questions about the content of the reviews or about the next steps, please just let us know by return email and we will schedule an appointment.

Update to Review Commons preprint posting policy
As of 1 August 2021, Review Commons requires all manuscripts to be posted as a preprint on either bioRxiv or medRxiv *prior to transfer to an affiliate journal*. In return, all the affiliate journals provide authors with scooping protection from the date of posting of the preprint (see detailed guidelines: https://www.reviewcommons.org/authors/). Please note that posting your reviews and your responses (refereed preprint) remains optional.

If you have not yet posted your manuscript as a preprint at the time of transfer to an affiliate, you will have the option to do so via the Review Commons submission system.

To access your manuscript online and proceed to the next steps, please use the link below:

Link Not Available

NEXT STEPS:

1. REVISION & RESPONSE TO REVIEWERS: There are 2 choices (see details: https://www.reviewcommons.org/authors/#submitting-to-affiliate):
- upload within *four weeks* a *preliminary revision* accompanied by a *revision plan* (download template: https://reviewcommons.org/templates/revision-plan.docx). Please use this template when submitting your preliminary revision.
- or, *request more time* via email to perform a *full revision* (download template: https://reviewcommons.org/templates/full-revision.docx). Please use this template when submitting your full revision.

2. POSTING THE PREPRINT: when submitting your revision, the system will require you to post it as a preprint if this has not yet been done. Posting the preprint is mandatory before transferring your manuscript to an affiliated journal. *Please note: only the preprint will be posted. Posting the reviews remains optional.*

3. REVIEWS ON BIORXIV or MEDRXIV: We strongly encourage the posting of your reviews on BioRxiv and MedRxiv. You will be able to instruct Review Commons to post the reviews and your response alongside your preprint. Please follow the instructions that are provided online on the submission screen.

4. TRANSFER TO AFFILIATE JOURNALS: When you submit the preliminary/full revision, you will be presented with a screen that allows you to select the affiliate journal to which you want to submit. Please note that the full set of reviews will be transferred alongside your response (revision plan or full revision) and the (preliminarily or fully) revised manuscript.

5. EVALUATION AT AFFILIATES: Upon transfer, the recipient journal will consider the manuscript as a formal submission and use the transferred reviews and your response/revision plan to make an initial decision. Note that while it is under consideration at an affiliate journal, the manuscript cannot be transferred to any other affiliates.

6. NEW ROUND OF TRANSFER: If the first journal declines, you will be directly informed by this journal. The manuscript will then be unlocked on the Review Commons site so that you can initiate up to two additional rounds of transfer.

Please consult our detailed Guidelines for further details on the process and policies of Review Commons at https://reviewcommons.org/authors. If you have any further questions, please contact me directly.

Thank you again for sending us your work. We hope you will find the reviews useful and that Review Commons helps to disseminate your peer-reviewed research faster and more efficiently.

Best wishes,

Sara Monaco
--
Sara Monaco, PhD
Managing Editor
Review Commons

Reviewer #1 (Evidence, reproducibility and clarity (Required)):

Insulators play key roles in organizing the genome into topological domains. Although diverse proteins are involved in insulator function, the mechanism of insulation is not fully understood. Recent association of DNA repair mechanisms, including presence of DNA double strand break (DSB) hot spots and phosphorylated histone H2A variant (γH2Av), a DSB repair marker, with key insulator proteins such as CTCF in vertebrates, suggests novel and important mechanism of genomic organization. However, evidence for causal relationship between insulator function and DNA repair mechanism remains to be strengthened.

The current study provided support that the link between insulators and DNA repair machinery may extend beyond the CTCF complexes. The gypsy insulator and its protein components are unique to Drosophila. Previous reports hey have associated mutants of gypsy components with genome instability and other phenotype relating to DNA repair. Using immunostain of Drosophila polytene chromosomes, the authors showed that the phosphorylated histone H2A variant (γH2Av) colocalizes with Su(Hw), Mod(mdg4) and CP190, three major components of the gypsy insulator, on both gypsy insertion sites and elsewhere in the genome. These γH2Av stains are diminished or abolished in mutants for the above insulator proteins. Analysis of public available ChIP-seq data sets further confirmed such colocalization between γH2Av and Su(Hw), and their colocalization with TAD boundaries. Okadaic acid, a drug known to block PP2A, a phosphatase that dephosphorylates H2Av following DSB repair, appears to intensify association between γH2Av and insulator proteins both on polytene chromosomes and in insulator bodies induced by hypertonic shock. Significantly, trans-heterozygous mutant of gypsy components and H2Av appears to disrupt the enhancer-blocking function of the insulator. The findings are significant because they strengthen the connections between diverse insulator subtypes and a hallmark of DNA repair machine. However, some concerns remain regarding interpretation of data and claims that need to be substantiated in a revised submission.

We thank the reviewer for recognizing the significance of the findings described in our paper and their contribution to further our understanding insulator function and mechanism in genome organization as well as the possible link with DNA repair. We wanted to emphasize though, that our findings in this work do not go beyond identifying a role for γH2Av in insulator function. These results do not support or reject that there is a functional association between DNA repair pathways and insulator function and the question remains a subject of research in my lab and elsewhere.

1. In the manuscript, the authors use the presence of γH2Av immunostaining interchangeably with the action of phosphorylation of H2Av. This is not supported. Addition of quantitation of γH2Av/H2Av ratio and its change under various mutant or treatment conditions are need to support this claim, as H2Av or even H2A occupancy could change under these condition. This important control should be provided for all major results.

We have revised the text of the manuscript to further clarify this point. We have now avoided referring to phosphorylation of H2Av as part of a mechanism and have replaced it, when possible, by phosphorylated H2Av through the text. We agree with the reviewer that phosphorylation of H2Av may be the subject of a complex regulatory mechanism, which most likely involves kinase and phosphatase pathways as well as histone chaperones that regulate the exchange H2A/H2Av in nucleosomes in response to changing developmental and environmental conditions, including

osmotic stress. Understanding these pathways would indeed be important to understand how they impact insulator function and genome organization.

The experimental control sample suggested by the reviewer (quantitation of γH2Av/H2Av ratio for every experimental condition assayed in the paper) would address the question of how H2Av occupancy and its phosphorylation state affects insulator function, which is not a question addressed in our paper. For this reason, we do not think that such experimental control is necessary for the interpretation of our current results. We believe that such experiments rather belong to a larger, more specific, future study assessing the mechanisms controlling H2Av phosphorylation in the context of insulator function, which we consider is out the scope of this work.

2. Genome-wide occupancy of different insulator types is often overlapping. For example, Mod(mdg4) and CP190 are known to colocalize with Drosophila dCTCF as well as to Su(Hw). This is seen in Figure 3F. Further, Mod(mdg4) and CP190 are also known to overlap at certain genomic locations with GAF and BEAF32, two other Drosophila-unique insulators. It would important to distinguish the contribution of γH2Av recruitment by different insulators. This could also explain why the Pearson correlation (PCC) between γH2Av and Su(Hw) is far lower than that for Mod(mdg4) or CP190. Computation analysis of genome wide colocalization of γH2Av with dCTCF, GAF and BEAF should also be performed. Colocalization of γH2Av with dCTCF and GAF in wild type and mutants could also be tested.

We agree with the point the reviewer is rising and we initially considered adding these analyses to Figure 3. For this work, however, we think it makes more sense to focus the analysis of global genomic distribution to the same proteins that showed strong colocalization with γH2Av in polytene chromosomes. Indeed, unpublished data in our lab shows that dCTCF and CP190 frequently colocalize with γH2Av in polytene chromosomes, but there is also clear absence of complete cololcalization, as is evidenced in Figures 6F and 6G where enrichment profiles of dCTCF do not match that of Su(Hw) and γH2Av.

These results are intriguing and we consider that further analysis of the relationships between γH2Av and other insulator proteins such as dCTCF, DP190, GAF and BEAF is necessary, but unwarranted for this report. To avoid confusion, we have removed the dCTCF and cp190 profiles from figure 6F and 6G in the final version of the manuscript.

3. The Okadaic acid disruption of PP2A function and its effects on γH2Av localization need to be better controlled to demonstrate target specificity. Disruption of such an abundant and broad-spectrum phosphatase may affect chromatin and nuclear organization via multiple different pathways that indirectly causes the observed changes on chromosome and in nucleus. For example, are there histones or chromatin proteins that are not modified by PP2A that can serve as a control?

The point of possible effects of OA specificity for phosphatase disruption is well taken. PP2A function may affect a wide range of phenomena. However, our results are a readout of the effects that phosphorylation levels have in the distribution of insulator proteins during normal conditions and

during osmotic stress. There is published evidence demonstrating PP2A is the phosphatase that specifically removes phosphorylation from γH2Av and our results indicate that effectively OA leads to a significant increase of phosphorylated H2Av.

Of course, we cannot rule out that dephosphorylation of other chromatin components may contribute to our observation. However, this only means that this possibility needs to be further explored. In our opinion, lack of a complete understanding of the pathways influencing γH2Av and its role in insulator function does not detract from our observation that levels of γH2Av appear to be positively correlated with levels of insulator proteins in the genome. We are not aware of any obvious control that could be applied to our experiments to clarify this point at this stage and consider that the analysis of possible pathways leading to our observation are out of the scope of the present work.

Minor points:

1. Consider merge Figure 4 and 5 a la Figure 2.

We understand the logic behind this suggestion, and we have merged figures 4 and 2 in this final version. However, we believe that Figure 5 represents a stand-alone result and is too big to be merged with the other two.

2. Typo: Figure 2B linear scan caption reversed

Thanks for noticing that. We have fixed the mistake.

3. Typo: Page 17 line 12, Figure S2B should be S1B.

Thanks for noticing this. We have fixed the mistake.

Reviewer #1 (Significance (Required)):

The findings reported here could be significant as they have potentially uncovered a novel and general mechanism of chromatin domain and genome organization. The manuscript is suited for a general audience in biological sciences.

We agree with the reviewer and appreciate his/her understanding and suggestions provided during the review.

Reviewer #2 (Evidence, reproducibility and clarity (Required)):

Phosphorylated Histone variant gamma-H2Av is associated with Chromatin Insulators in Drosophila by Simmons JR et al.

This manuscript shows a relationship between phosphorylated H2Av (gamma-H2Av) with gypsy insulator components Su(Hw), Mod(mdg4)67.2, and CP190 in Drosophila melanogaster. They show that insulator proteins Su(Hw), Mod(mdg4)67.2, and CP190 co-localize with gamma-H2Av through immunofluorescent microscopy (Figure 1) and that gamma-H2Av is present at gypsy insulator cites in the yellow2 and cut6 alleles (Figure 2) in polytene chromosomes respectively. In Figure 2, they also show altered co-localization of H2Av with proteins Su(Hw), Mod(mdg4)67.2, and CP190 at the yellow2 and cut6 alleles in mutant backgrounds mod(mdg4)u1, su(Hw)e04061, and cp190P11/H31-2. They find that in these mutant lines gamma-H2Av levels decrease in su(Hw)e04061 and cp190P11/H31-2 with the exception of mod(mdg4)u1 background (Figure 4) and that these insulator components are dependent on each other in order to co-localize with gamma-H2Av (Figure 5). In addition, the authors observe a co-enrichment of Su(Hw) at gamma-H2Av ChIP peaks and a co-enrichment of gamma-H2Av and Su(Hw) at called TAD boundaries in publicly available S2-cell datasets (Figure 3). They also observe that under stress conditions (hypertonic conditions), gamma-H2Av co-localizes with insulator bodies and that phosphatase inhibition is sufficient at blocking insulator bodies dispersion upon recovery (Figures 6,7). Ultimately they provide a model in which gypsy insulator components mediate gamma-H2Av and a putative role of gamma-H2Av in insulator activity by exhibiting that reduction of dose of H2Av in a sensitized (su(Hw)) background alleviates gypsy insulator phenotypes observed at yellow2 and cut6 alleles (Figure S3).

Overall, the findings are very interesting. However, there are a number of points that would significantly strengthen the manuscript, that I will review below.

Major comments:

1. The fly lines used throughout the manuscript are either not sufficiently described, or only described in detail after being first mentioned, resulting in a non-optimal flow that complicates the interpretation of results by the reader.

We have edited the text adding fly stocks information to all the genetic experiments as they are described in the results.

2. For examinations involving mod(mdg4)u1, the use of mod(mdg4)T6 in concurrence would support the results further, especially when analyzing levels of gH2Av in this background (Figure 4).

Unfortunately we lost our mod(mdg4)T6 stock in the laboratory and cannot perform this experiment right away. However, we did not consider this experiment necessary before submitting our manuscript for publication for the following reasons:

I: We have verified the results linking phosphorylation of H2Av to Su(Hw) by performing the experiments with homozygous as well as trans-heterozygous flies. This results support the conclusion that normal levels of phosphorylation of H2Av depend on the Su(Hw) protein, and do not depend on an unrelated unknown second site mutation.

*II: We did not initially seek to use modT6 alleles because **both T6 and U1 are null alleles** of the mod(mdg4)67.2 isoform. We reasoned that the observed result is unlikely the consequence of a secondary site mutation in the modU1 stock. Such second site mutation would have to behave as modifier enhancer of H2Av phosphorylation in a mutant background (U1) that suppress H2Av phosphorylation. This would have to be the case if results with modT6 happened to be opposite form U1. This would be an interesting finding but very unlikely and would be difficult to interpret withing the present work. Additionally further analysis of such finding would require additional time-consuming experiments that would be outside the scope of this manuscript.*

Our interpretation of this results is that phosphorylation of H2Av requires Su(Hw) but is independent on Mod(mdg4). We consider the specific mechanisms of H2Av phosphorylation as well as the responsible kinase and roles of different insulator proteins in its association with Su(Hw) very interesting and as part of future research in our lab.

3. In Figure 1, the methods section fails to explain better how quantification of the fluorescent signal is made. It's generally not clear if when looking at the whole genome this means the whole length of polytene chromosomes is analysed, or whether randomly selected regions are used.

The analysis involves full polytene chromosomes, and this is done by selecting the entire nucleus using DAPI staining as ROI and calculating the intensity of immunostaining in the selected region. We have clarified this in the text now.

4. Figure 2 should be further supported by more quantitative intensity measures at the y2 and ct6 loci. In addition, it is not clear if the authors only looked at one polytene site/one organism or multiple.

Multiple nuclei and larvae were used in this experiment. However, quantifying the intensity at the y2 and ct6 loci specifically, would be challenging given that in the su(Hw) mutant the reference signal corresponding to both loci would not be present. Additionally, the precision of the mapping using polytene bands as reference with a quantitative purpose may lead to questionable results. The goal of this experiment was to confirm that the Su(Hw)-γH2Av association observed for global genome sites is also true for Gypsy insertions, which carry the canonical Su(Hw) gypsy insulator. Colocalization of y2 and Ct6 mutations with γH2Av in this samples confirms this prediction.

5. It would be useful if the authors would submit the code used to perform the analyses in Figure 3 to a public repository (e.g., GitHub). In particular, a better description of the panels is necessary. There is no mention of peak strength used for example for panel A. The scale bar for the colors used in missing for both A and C. In G, there is no discernible visual difference which can be seen in overlap between H2Av Het/Hom as compared to gamma-H2Av signal in one boundary compared to the other as stated in the text (pg. 14-15). The author's statement as to whether γH2Av would be non-nucleosomal is not supported in the data presented and should be clarified.

Graphical representation of the Data in Figure 3 was obtained using seqMINER 1.3.4. which is a ChIP-seq data interpretation platform that is publicly available in the web, as we indicate in M&M. Peaks were not selected for quantitative analysis in any of the panels of figure 3.

Heatmaps generated with seqMINER use sequence enrichment values extracted from Bam files and are not based in peak strengths. Additionally, heatmaps generated by seqMiner do not produce a color scale, but darker color represents higher enrichment along a sequence. Peaks were only used in Figutre 3A, where ChIP-seq data for Su(Hw) is centered at γH2Av peaks where γH2Av peaks were produced by generating a BED file Using MACs2 callpeak from Galaxy.

We have further clarified this in Material and Methods and we have addressed the non-nucleosomal statement in the text by specifying that we do not have direct experimental evidence supporting the claim that some of the γH2Ave is non-nucleosomal. We only speculate this could be the case given the enrichment distribution profile at certain sites and the absence of unphosphorylated H2Av in insulator bodies.

6. Figure 5 doesn't include Mander's measurements. In panel F, results from control flies would help in this comparison, as well as appropriate statistical testing. There is no discussion as to why in the mutant CP190 background, Mod is not looked at.

Given the lack of colocalization between insulators and γH2Av in the mutants we did not think Mander's measurements would be necessary. However, we added Modifier data in CP190 mutant background in the final version of the manuscript. These data now include modifier data in CP190 mutants and in CP190 mutants treated with Okadaic acid

7. In Figure 6D, data from colocalization of non-phosphorylated H2Av with gamma-H2Av would be a good control to show inverse relationship under stress shown in 6G, and colocalization with Su(Hw) would be a good negative control.

To clarify the relationship between signals from anti-H2Av and anti-γH2Av antibodies we have added western blot data to Figure 5, showing that both antibodies are highly specific. Co-Immunostaining using both antibodies show no signal overlap (anti-H2Av does not recognize γH2Av and anti-γH2Av does not recognize H2Av). Also see response to Reviewer 3 #3

8. Figure S3 is perhaps the most important proof of concept in showing gamma-H2Av playing a part in gypsy insulator function and should not be in the supplements.

We have moved the old figure S3 to the main section as Figure 7.

9. A coIP of gamma-H2Av interaction with insulator components would be necessary in order to support the model provided in Figure 8.

As suggested by the reviewer, we have performed several CoIP experiments during this time, but we were unsuccessful in the CoIP of γH2Av and Su(Hw) under our experimental conditions. However, we think our protocol needs to be significantly improved before we can conclude that the result is negative. Further ChIP-seq and CoIP approaches are planned for future experiments addressing this question in a new work that will address this problem in more detail.

We have addressed this issue in the text by specifying in our model figure (figure 8) that, based on our findings, we cannot necessarily infer that a direct interactions between γH2Av and Su(Hw) exists. We have pointed out that interactions may occur between γH2Av and any of the components of the Su(Hw) insulator complex. We have also discussed the alternative possibility that the interactions between γH2Av and the insulator may be mediated by weak multivalent interactions. We based this speculation in that we have recently published a paper describing that insulator proteins, including γH2Av have liquid-liquid phase separation properties in vivo and that they are able to form liquid-droplets.

Additionally, we have further strengthened the evidence of colocalization of both Su(Hw) and γH2Av with boundaries in chromosomes by providing additional examples in supplementary figure 1 and in new panels in Figure 4.

10. ChIP data for S2 cells of gamma-H2Av, H2Av, and Su(Hw) in osmotic stress conditions would help support claim of dissociation/creation of granules rather than re-localization.

We agree with the reviewer it is very important to produce results interrogating osmotic stress as well as with different background mutations and conditions with ChIP-seq data in S2 or Kc cells. However, we believe microscopy data presented here and in previous publications strongly support the notion that γH2Av associates with insulator bodies during osmotic stress as well as during normal physiological conditions, which is the main conclusion of this work. ChIP-seq data for γH2Av, H2Av, and Su(Hw), among others, is necessary to quantify he levels at which both Su(Hw) and γH2Av associate with different compartments in the nucleus, particularly insulator body and chromatin compartments during osmotic stress, but these are experiments we are planning for future work.

11. Figure S1 could be better labeled to include treatment with OA outside of figure legend.

Okadaic acid treatment and molarity is now indicated in each panel of the figure

12. Figure S2 would better support restoration through phosphatase inhibition if previous results were included in S2F to also show the extent of restoration. This could also be better labeled in the figure itself as to be under phosphatase inhibition condition.

We understand the point raised by the reviewer, however given the space limitation in the figure we have not found the necessary space required to properly provide the ability to compare treated and untreated chromosomes. We consider that the reader can refer to untreated chromosomes shown in previous figures. Also, linescans and PCC values can be compared with the previous figures showing untreated chromosomes.

We have added a new quantitative analysis of modifier immunostanining in the background of CP190 mutants which was missing in the previous version of the figure.

We find difficult to add the OK treatment label to each panel in figure S3. Because the entire figure S3 consist on Okadaic acid treated samples and this is indicated in the figure legend we have decided to keep the figure without additional labels,

Minor comments:

13. Generally, the figures/results could be reorganized to flow better. The first figure could be followed by a combined figure made of Figures 4 & 5 showing the general effects of mutant backgrounds on H2Av, then Figure 2 where specific known insulator sites are examined. This can allow for a better flow to then analyze co-localization in public ChIP data and dynamics under normal and stress conditions, ultimately showing reduction in cut6 yellow2 phenotypes in double hemizygous flies.

We have merged figures 4 and figure 2 in a single new figure 2. As a result, most figures have been renumbered and the flow of the paper now has changed in a way that we think helps understanding by the reader.

14. The organization of Figure 1 is hard to follow in the text and the figure legend. First, panel A should be the combined whole image of polytene chromosomes with various gypsy components and gamma-H2Av. Panel B can then be the magnified insets and panel C can be the line-scan intensities. Panel D can then be the PCC and Mander's calculations made based on whole genome measurements (which need to be better described as to how this was carried out and what these measurements mean). This will allow for a better descriptive legend of what each panel is exhibiting.

We have considered this suggestion, but found the final combination of panels, in our opinion, actually more confusing and harder to follow and understand. We have made improvements in the figure as suggested by the reviewer in point 18.

15. Figure 4 seems better fit as a subsection in Figure 2 or Figure 5.

see comments from point 13

16. For Figure 3, examples loci from the clusters should be provided. I think boundary strength should also be included in this analysis. There seems to also be a third cluster which is not discussed in panel C.

We have added a new supplementary figure providing new examples for clusters 1 and 2 and added three examples for cluster 3. We are grateful for the suggestion of the reviewer to analyze how γH2Av changes with changes in boundary strength. We now provide evidence that γH2Av enrichment along the chromosome correlates with boundary strength. We used the concept of TAD-separation score developed by the Manken group and show that γH2Av is enriched at sites with los TAD-separation scores, which correspond to TAD boundaries. In figure 4 we provide new results showing that strong boundaries are enriched in γH2Av.

17. The first few sentences in the introduction seem detached from the actual topic of the paper and its findings. They imply that the work will be centered around genome conformation data rather than the association of gamma-H2Av and gypsy insulator components.

We see the point of the reviewer and have modified the text in the introduction accordingly.

18. In figure 1, a better distinction than the use of arrows and arrowheads could make the plots more easily understood.

We have modified the figure to make sure the it is easier to understand, using new formatted arrows with numbering to relate to the line diagrams.

Reviewer #2 (Significance (Required)):

This work presents a conceptual advance that establishes a link between gamma-H2Av and chromatin insulators (and by proxy, therefore, a link with chromatin three-dimensional organization). The link in interesting and seems to be supported by the data, although additional clarifications are needed as described above.

The work adds to the increasing body of literature examining the role of different molecular mechanisms in establishing genome organization and its functional relevance, and it is likely to be interesting for audience working in the fields of epigenetics and development.

My field of experience is: chromatin conformation, development.

Reviewer #3 (Evidence, reproducibility and clarity (Required)):

Summary:

Presented research demonstrates a colocalization of Su(Hw) architectural protein and its molecular partners with the sites of γ-phospho-H2Av histone mark (a well-described marker of double-strand breaks) at the polythene chromosomes. Authors demonstrated a significant decrease in γ-phospho-H2Av distribution at the polythene chromosomes in Su(Hw) mutants. They also showed a presence of Su(Hw) in γH2Av sites in Kc cells (using previously published ChIP-Seqs of Su(Hw) and γH2Av from different studies). Upon osmotic stress γH2Av signal was shown to decrease in a nucleus of S2 cells and aggregate forming Su(Hw)-enriched cytoplasmic bodies. The authors suggest that Su(Hw) is important for H2Av phosphorylation and γH2Av plays a role in the insulator function of Su(Hw).

Major comments:

1) The most questionable statement in the research is a claim of a presence of γH2Av mark in Kc cells in the untreated conditions. There is no enough evidence for that. Normally, cells need the specific stress treatment (irradiation or heat shock) to accumulate detectable level of γH2Av. For example, the study authors used to obtain γH2Av ChIP-Seq data demonstrated a complete absence of γH2Av signal in the untreated Kc cells and its significant elevation upon PR-Set7 knockdown (Fig 3 of PMID: 27131378).

The point the reviewer is making here relates to the microscopic analysis of γH2Av fluorescence intensity, which has traditionally been applied to the analysis of DNA double strand break repair mechanisms. However, no data is available demonstrating at the molecular level that there is no significant γH2Av present in untreated diploid cells.

It is true that the large foci and strong signal intensity that appears in diploid cells labeled with anti-γH2Av only occurs after induction of DNA damage (as shown in the paper referenced by the reviewer analyzing PR-Set7 knockdowns). Untreated cells do not show such large foci or intense immunostaining signals, appearing as if there is no γH2Av present. This is true for both γH2AX in vertebrates and γH2Av in Drosophila. Experimental settings in such experiments, however, demand high contrast between cells undergoing DNA damage repair and cells that don't. Under such conditions, and depending on the sensitivity of the antibody, basal levels of phosphorylated H2Av may remain undetected. Figure 6 (A and B) in our paper demonstrates that untreated S2 cells display a significant γH2Av immunostaining signal that represents the γH2Av associated to chromatin under normal conditions.

Moreover, molecular data in the same paper referenced by the reviewer contradicts his/her conclusion: Supporting our immunostaining data in polytene chromosomes and in S2 cells, ChIP-seq using the same anti-γH2Av antibody we use reveals a consistent and specific enrichment genome profile of phosphorylated γH2Av under normal conditions.

Our work actually shows that there is a significant population of phosphorylated H2Av associated to chromatin in untreated cells with a function that is not likely related to DNA repair. This population of γH2Av is normally present in cells in the absence of DNA damage. The enrichment of this γH2Av in chromatin is less than that corresponding to the γH2Av normally involved in DNA damage repair, but is significant and produces a consistent ChIP-seq genome profile.

We base this argument on our own observations analyzing the published ChIP-seq data and our own polytene chromosomes and S2 cells immunostainings. This argument is also supported by a number of published reports that involve the drosophila H2Av in processes such as heterochromatin formation and in the development of the immune system (Swaminathan et al. 2005. Genes Dev. 19(1):65-76; Grigorian et al. 2017. Development, 144(8): 1441–1449). Whereas it is true that these examples do not specifically differentiate H2Av from γH2Av, another publication demonstrates that γH2Av plays a role in regulation of transcription and is found associated to active promoters (Kotova et al. 2011. PNAS 108, 6205-6210).

Supporting these observations, our analysis of ChIP-seq data found that γH2Av is enriched at promoters (not shown in our paper) and at Su(Hw) sites, including TAD boundaries.

Some γH2Av peaks authors called in the untreated Kc cells using GSE73668 may be a result of inappropriate ChIP-Seqs normalization (GSE73668 does not provide the Inputs for the ChIP-Seq. Probably, the authors had to use third-party Inputs to call the γH2Av sites which led to some errors in normalization).

We actually normalized the Chip-seq data using a control Chip-seq samples coming from the the same lab (MacAlpine) in Kc cells under the same control conditions expected for the data presented in our paper (reference). Normalized data show that the peaks associated to Su(Hw) remain unchanged. We did not show this in the paper given that the data was published and the control data came from a different paper, but we will be happy to share this analysis if requested or include it in the paper if judged necessary.

Supporting this, γH2Av ChIP-Seqs in salivary glands (GSE55932, PMID: 25437540) shows no peaks of γH2Av at the Su(Hw) sites both at the BX-C and eve loci (loci authors used to co-localize γH2Av and Su(Hw)). Moreover, in salivary glands γH2Av signal does not accumulate in the form of individual peaks, but shows a wide distribution over chromoso. mes.

We were aware of the existence of γH2Av Chip-seq data in salivary glands and decided against using it in our work. GSE55932, PMID: 25437540 uses a rabbit anti-γH2Av antibody from Rockland. We had used the same antibody to analyze the effect of su(Hw) mutations in the Drosophila female germline (Chromosoma 2020 Dec;129(3-4):255-274). In our hands, this antibody is sensitive only to high levels of phosphorylation such as those produced in response to global DNA damage. Our experiments of immunostaining in wildtype ovaries or salivary glands never showed any significant immunofluorescence signal, which makes us very skeptical about the validity of the global distribution of γH2Av shown by the published ChIp-seq data in salivary glands.

Fluorescence microscopy experiments described in our manuscript use a mouse monoclonal anti-γH2Av, which is the same antibody used to generate the Kc cells ChIP-seq data in the MacAlpine publication. The mouse monoclonal anti-γH2Av that we used was developed and thoroughly characterized by the Hawley lab (Cathleen et al 2013 G3 Genes|Genomes|Genetics, Volume 3, Issue 9 1539–1543. Referenced by 42 publications) in order to obtain a more specific and more reliable anti-γH2Av antibody than the previously existing Rockland rabbit anti-γH2Av. The monoclonal anti-γH2Av is also available for purchase at the Developmental Studies Hybridoma Bank from the university of Iowa.

Given that our results using this antibody in polytene chromosomes are completely consistent with our S2 cells data and with the Kc ChIP-seq data from MacAlpine's lab, we questioned the validity of

using the salivary gland ChIP-seq for interpretations of global γH2Av genomic distribution data. This question does not take away the results shown in the referenced paper aligning γH2Av enrichment with under replication sites, which suggests the Rockland antibody seems to work well with the bulk concentrations of γH2Av associated with DNA damage. Please note that, to my knowledge, no microscopy polytene data has been published showing γH2Av distribution in polytene chromosomes using the Rockland rabbit anti-γH2Av antibody, including PMID: 25437540 and PMID: 26051888, which only show ChIP-seq data in salivary glands and follicle cells respectively. The only data on anti- γH2Av in polytenes was performed by the Zimulev lab with a rabbit anti-γH2Av developed by the Glasser lab (Madigan et al. 2002. Nucleic Acids Res. 30(17): 3698–3705) rendering data that is not comparable with the data obtained with the monoclonal anti-γH2Av in polytenes and in Kc cells.

2) The very different story is a presence of γH2Av mark in normal polytenized chromosomes, which was indeed demonstrated (PMID: 25437540, PMID: 26051888). The reason is that polytenized chromosomes have multiple areas of underreplication filled with stalled replication forks. Previously, it was found that the γH2AX mark (the mammalian analogue of γH2Av) not only marks double strand breaks, but is also introduced into stalled replication forks to rescue them (PMID:22787276). So, presence of γH2Av indeed correlates with the underreplicated regions and may mark stalled replication forks (PMID: 25437540).
The role of Su(Hw) in introduction of γH2Av into salivary glands polythene chromosomes is indeed very interesting. But to elucidate the exact role of Su(Hw), more detailed colocalization of the signals is needed, using ChIP-Seq from the salivary glands (better in control and mutated backgrounds - to demonstrate the loss of Su(Hw) and γH2Av signals upon mutation).
I would suggest to the authors to deepen their story about γH2Av in salivary glands and delete the part about γH2Av in Kc cells as there is not enough evidence for this.

We have argued through the responses to reviewer 3 that this major critique is not supported by the evidence. For example, based on the interpretations of the reviewer, it would have to be argued that the colocalization of Su(Hw) with γH2Av in salivary glands is due to undereplication or to any other DNA break-related mechanism, specific of salivary glands. However there is no evidence in the literature or in our own work directly linking Su(Hw) with underreplication or with DNA repair for that matter. Furthermore, the evidence of colocalization of Su(Hw) with γH2Av we observed by microscopy is not supported by salivary gland γH2Av ChIP-seq, while it is strongly supported by the Kc cells ChIP-seq data we present.

3) Both describing the ChIP-Seq results and osmotic insulator bodies authors claim an existence of some "non-nucleosomal γH2Av", as they do not detect the H2Av signal at the sites of γH2Av. These results look unexpected and make suspect antibodies of insufficient specificity. In the Methods authors do not provide the description of anti-H2Av antibodies but it is highly unlikely that they can not bind γH2Av histone. That is more likely and unfortunate that anti-γH2Av antibodies cross-reacts with some other phosphorylated protein. The specificity of γH2Av antibodies needs further confirmation. The confirmation option could be Co-IP with the anti-Su(Hw) antibodies. That would confirm Su(Hw) interaction with γH2Av but not with other protein.

The reviewer is right when questioning the validity of the antibodies and their specificity when performing the experiments described in the paper. We have tested the specificity of the antibodies in my lab using western. Western results are shown now in Figure 5 revealing two bands very close to each other representing H2Av and γH2Av, each detected using fluorescence with a different

secondary antibody, demonstrating the high specificity of both antibodies. Difference in band size results from the phosphorylation in the γH2Av slightly reducing its migration rate in the gel.

This result demonstrates that the antibodies we used are highly specific and that the H2Av antibody does not crossreact with the phosphorylated version of the same γH2Av protein. We did inquire the company that produced the antibody (Active Motif) and we were told it was raised against a peptide that was unique to H2Av as antigen corresponding to the C terminal tail (the specific peptide was commercially protected and was not made available to us). We concluded that the antigen peptide included the S137 site and that the antibody anti H2Av fails to recognize the phosphorylated version of the antigen in γH2Av.

Antibody specificity further confirms that insulator bodies contain γH2Av but not H2Av, and that the γH2Av is unlikely nucleosomal at insulator bodies.

4) In the Abstract authors claim "Together, our results indicate a novel mechanism linking the H2A variant γH2Av to insulator function". But they do not provide experiment to confirm that statement. Indeed, their experiments on measuring the Su(Hw) insulating activity in doubly heterozygous for h2av and su(Hw) mutants confirm mild but significant effect of h2av gene on Su(Hw) activity. But this result does not support the participation of exact γH2Av mark in insulator function.

We agree with the reviewer in that we have to be more careful with the conclusions and statements that are supported by our data. We have revised the paper to address this and other issues brought up by reviewers. In this example we have changed "Together, our results indicate a novel mechanism linking the H2A variant γH2Av to insulator function" for "Together, our results indicate a novel mechanism linking the H2A variant to insulator function"

Minor comments:

1) In the Discussion authors claim "in a number of instances where γH2Av participates in regulation of chromatin structure (Swaminathan et al., 2005)". But in the provided study, there is no mention of γH2Av under normal conditions - only H2Av.

We agree with this remark and we have reviewed the text to clarify this important distinction. However, Kotova et al. (2011) link H2Av with γH2Av in its role on transcription regulation. We have cited this paper in this context to support the notion that γH2Av, in addition to DNA repair, has also roles in transcription, which is in line with our findings that is also found in boundary elements in the genome.

2) Unfortunately, the role of Su(Hw) in the incorporation of γH2Av into polythenized tissues still looks very controversial. In their previous work authors showed that su(hw) mutation leads to increase in γH2Av signal in early stage ovarioles (PMID: 33140220). But this research provides evidence that su(hw) mutants on the contrary lack γH2Av signal in salivary glands. Unfortunately, authors do not

discuss this discrepancy. It appears that Su(Hw) influences genome amplification rather than being involved in the cascade leading to γH2Av introduction.

We agree with the reviewer that there are questions that still need to be answered about the relationship between su(Hw) and DNA repair and this will be a future focus of research in our lab. However, we do not believe that our previous results are inconsistent with the results presented in this paper. In our previous paper we presented evidence that lack of Su(Hw) leads to DNA damage, both in ovaries and in developing neurons, which explains the increase in γH2Av as shown in ovaries. However we do not have evidence that lack of Su(Hw) induces DNA breaks in salivary glands. Our hypothesis is that phosphorylation of H2Av in DNA repair and in insulator function follows different pathways. This hypothesis requires further experimentation and will be tested in the future

3) In the Methods authors referenced SRX1299942 (γH2Av SRA (Li et al., 2016)) as ChIP-Seq they used to colocalized with Su(Hw) but this exact SRX contain γH2Av ChIP-Seq performed upon PR-Set7 RNAi conditions. Maybe that's a typo. Obviously, ChIP-Seqs performed in different conditions can not be colocalized.

I apologize for this. It was a typo and it has been corrected in the manuscript accordingly (substituted by SRX1299943)

Reviewer #3 (Significance (Required)):

Proposed study contains two major lines of investigation: description of Drosophila Su(Hw) architectural protein role in γH2Av deposition in the (1) cultural cells and the (2) polytene chromosome of salivary glands. The former is problematic, as previously no significant γH2Av signal was found in cultured Drosophila cells under normal untreated conditions. Second, the Su(Hw) role in γH2Av deposition in polytene chromosomes is an interesting question. Unfortunately, the authors used only the polytene chromosomes staining to prove the joint distribution of Su(Hw) and γH2Av. Authors need to provide more precise data describing Su(Hw) and γH2Av co-localization in polytene chromosomes by ChIP-Seqs made of Drosophila salivary glands (both in control and mutant condition).

We have argued during the response to reviewer #3 that the findings associated to γH2Av ChIP-seq data in Kc cells cannot be dismissed as artefacts and require an interpretation. We present analysis of these data in our manuscript showing that γH2Av is enriched at Su(Hw) sites. This observation remains true even after normalization with a control sample. Overall, these conclusions are supported by our observations in salivary gland polytene chromosomes, in S2 cells during osmotic stress and because double heterozygous flies present a partially rescued phenotype in gypsy insulator induced mutants. In the response we have also expressed our concern that existing salivary gland ChIP-seq data may not be providing a representative profile of the γH2Av genomic

distribution, given that the antibody utilized in the published ChIP-seq failed to produce a significant signal in immunostaining experiments using fluorescence microscopy in polytene chromosomes in our lab.

Our therefore consider our results significant. Our findings indicate that phosphorylated H2Av plays a role in insulator function. Findings presented in our manuscript have unveiled a new role of γH2Av in genome organization and we believe the warrant access to the scrutiny by researchers in the field by publication, to be either confirmed or challenged by future independent research.
